# Cardiovascular Effects of Cumulative Doses of Radioiodine in Differentiated Thyroid Cancer Patients with Type 2 Diabetes Mellitus

**DOI:** 10.3390/cancers14102359

**Published:** 2022-05-10

**Authors:** Adina Elena Stanciu, Marcel Marian Stanciu, Anca Zamfirescu, Dan Cristian Gheorghe

**Affiliations:** 1 Department of Carcinogenesis and Molecular Biology, Institute of Oncology Bucharest, 022328 Bucharest, Romania; 2 Electrical Engineering Faculty, University Politehnica of Bucharest, 060042 Bucharest, Romania; marcel.stanciu@upb.ro; 3 Department of Radionuclide Therapy, Institute of Oncology Bucharest, 022328 Bucharest, Romania; ancacvi@gmail.com or; 4 ENT Department, University of Medicine and Pharmacy Carol Davila Bucharest, 050474 Bucharest, Romania; gheorghe.dancristian@gmail.com

**Keywords:** differentiated thyroid cancer, type 2 diabetes mellitus, ^131^I, LVEF, platelet counts

## Abstract

**Simple Summary:**

The cardiovascular effects of radioiodine (^131^I) therapy on people with differentiated thyroid cancer (DTC) and concomitant type 2 diabetes mellitus (T2DM) are unknown. To the best of our knowledge, this study is the first of its kind. Our aim was to assess the relationship between the left ventricular ejection fraction (LVEF) and high cumulative ^131^I doses in DTC/−T2DM and DTC/+T2DM female patients. In the DTC/−T2DM group, LVEF was negatively associated with high cumulative doses of ^131^I. This association did not exist in patients with DTC/+T2DM.

**Abstract:**

Radioiodine (^131^I) therapy for differentiated thyroid cancer (DTC) involves exposure of the whole body, including the heart, to ionizing radiation. This exposure to the subsequent risk of heart disease is uncertain, especially in patients with DTC associated with type 2 diabetes mellitus (DTC/+T2DM). The current study aimed to assess the relationship between left ventricular ejection fraction (LVEF), high cumulative ^131^I dose, and peripheral blood parameters in patients with DTC/−T2DM and DTC/+T2DM. The study enrolled 72 female patients with DTC/−T2DM and 24 with DTC/+T2DM who received cumulative ^131^I doses above 150 mCi (5.55 GBq). LVEF was lower in patients with concomitant T2DM than those without (*p* < 0.001). The cumulative ^131^I dosage was inversely correlated with LVEF only in DTC/−T2DM patients (*r* = −0.57, *p* < 0.001). In the DTC/+T2DM group, LVEF was negatively associated with absolute platelet count (*r* = −0.67, *p* < 0.001) and platelet-to-lymphocyte ratio (*r* = −0.76, *p* < 0.001). Our results demonstrate that exposure to high cumulative ^131^I doses has different cardiovascular effects in DTC/−T2DM and DTC/+T2DM.

## 1. Introduction

According to the International Diabetes Association, 1 out of 10 adults live with diabetes worldwide (537 million) [1,2]. The number of people diagnosed with diabetes will rise to 643 million by 2030 and 783 million by 2045 [1,2]. A causal association between type 2 diabetes mellitus (T2DM) and various cancers (liver, pancreatic, breast, and endometrial cancer) was confirmed in a meta-analysis comprising 32 million people [2]. Moreover, this association has increased for prostate, colon, and gallbladder cancer in the last decade [3].

Recent data have shown that patients with well-differentiated thyroid cancer (DTC) treated with total thyroidectomy have an increased risk of up to 40% of developing T2DM, regardless of age [4]. This risk is also increased with low as well as high doses of postoperative levothyroxine [5]. Basically, total post-thyroidectomy suppression of thyroid-stimulating hormone (TSH) may have detrimental effects on glucose homeostasis in patients with DTC [4]. According to the guidelines developed by the European and American Thyroid Associations (ETA and ATA) [6,7], the treatment of DTC consists of thyroidectomy followed by TSH suppression therapy and radioactive iodine (^131^I) targeted intracellular therapy [6,7]. Strong uptake of ^131^I into the thyroid bed is a prerequisite for postsurgical thyroid remnant ablation. ^131^I therapy for DTC involves exposure of the whole body, including the heart, to ionizing radiation. Many previous studies have focused on stroke and cerebrovascular diseases in patients with benign thyroid disease or thyroid cancer who received a relatively low cumulative ^131^I dosage [8,9]. The effect of exposure to high cumulative doses of ^131^I on the subsequent risk of heart disease is uncertain.

Blood cell interactions are essential in the pathophysiology of inflammation and immune responses in DTC patients [10]. These interactions have many facets, and it is often difficult to distinguish the specific roles of each cell type in response to high cumulative doses of ^131^I. It is even more difficult when DTC is associated with an inflammatory condition, such as T2DM. The most common circulating white blood cells are neutrophils, which play important roles in tumor cell proliferation or the development of atherosclerosis, leading to cardiovascular and obesity-related diseases, such as T2DM [11]. Chronic inflammation in T2DM is characterized by a prothrombotic state caused by the mutual activation of neutrophils and platelets. Platelet–neutrophil complexes enhance platelet activation and thrombus formation [12]. The role of neutrophils and platelets in mediating radiation response remains controversial because tumor-associated neutrophils or platelets can have both pro-tumor and anti-tumor effects. Wisdom et al. [13] showed that neutrophils promote resistance to radiation therapy, suggesting therapies designed to lower neutrophils during radiotherapy. The number of inflammatory cells (such as platelets, neutrophils, lymphocytes) and the ratios between them, such as the neutrophils-to-lymphocytes ratio (NLR) and platelets-to-lymphocytes ratio (PLR), can reflect the systemic inflammatory status and predict clinical outcomes in cancer patients [14,15]. NLR and PLR have been associated with clinical outcomes in patients receiving selective internal radiation therapy [15].

Although people with T2DM are known to have a 2 to 4 times higher risk of cardiovascular morbidity and mortality than people without diabetes, the risk of patients diagnosed with DTC coexisting with T2DM (DTC/+T2DM) is unknown. Epidemiological studies have shown that the relative risk of vascular events is more significant in women in long-standing diabetes mellitus [16]. Patients with DTC/+T2DM fall into this profile. The cardiovascular effects of ^131^I therapy on people with DTC/+T2DM are unknown. To the best of our knowledge, this study is the first of its kind. Our aim was to assess the relationship between high cumulative doses of ^131^I, echocardiographic indices of left ventricular function, and peripheral blood parameters in DTC/−T2DM vs. DTC/+T2DM patients.

## 2. Materials and Methods

### 2.1. Patients and Study Protocol

This retrospective study from a single institution included 72 female patients with DTC/−T2DM (mean age, 57.9 ± 8.7 years) and 24 female patients with DTC/+T2DM (mean age, 61.1 ± 7.2 years) undergoing total thyroidectomy with dissection of central lymph nodes and lymph node of the affected side and ^131^I therapy. The enrolled patients were treated between 2015 and 2020 in the Department of Radionuclide Therapy of the Institute of Oncology Bucharest with a cumulative oral dose of ^131^I sodium iodide ThyroTop higher than 150 mCi (5.55 GBq). This cumulative dose was administered over a mean period of 53 months in 4 cycles of ^131^I therapy. ThyroTop^131^ is a radiopharmaceutical purchased from the Institute of Isotopes Co., Ltd. (IZOTOP), Budapest, Hungary. The administered activity (“dosage”) of ^131^I in this cohort of patients was based on clinical and diagnostic imaging following the recommendation from the ETA and ATA guidelines [6,7] in compliance with safety measures [17].

The patients were included in the study based on the following criteria: (i) age between 40 and 70 years; (ii) non-smokers; (iii) access to the patient’s medical and drug history; (iv) none of the patients had an acute or chronic infection, pulmonary, hepatic, or renal impairment; (v) none of the patients had a history of cardiovascular disease (heart failure, acute coronary syndrome, symptomatic valvular dysfunction, or cardiomyopathy); (vi) no patient had poorly controlled diabetes; (vii) none of the patients had ongoing treatment with steroidal or non-steroidal anti-inflammatory drugs (known to affect the NLR and PLR value) [18]. Only women were enrolled to avoid intersex variations.

Demographic information (age, smoking status, hypertension, body mass index (BMI)), cumulative ^131^I dosage/patient, the daily dose of levothyroxine/patient, echocardiographic indices of left ventricular function, blood count, and serum biochemical parameters (total cholesterol, triglycerides, total lipids, alkaline phosphatase (ALP), ionized calcium (Ca^2+^)) were collected from medical records and correspond with the most recent hospitalization (6 months after the last therapeutic dose of ^131^I). All the patients diagnosed with hypertension included in the study were treated with blood-pressure-lowering medication, and their blood pressure was within the normal range. Left ventricular ejection fraction (LVEF) was measured in the apical two- and four-chamber views using Simpson’s biplane method. For clinical interpretation of the data, myocardial dysfunction was defined as an LVEF < 54%, according to the range of normal LVEF in the general female population [19]. The NLR and PLR were calculated by dividing the absolute neutrophil or platelet count by the lymphocyte count.

The study was carried out respecting the principles outlined in the Declaration of Helsinki and was approved by the Institute of Oncology Bucharest ethics committee (No. 15140/10.09.2019). Informed consent was obtained from all patients.

### 2.2. Statistics

Microsoft Office Excel 2007 SP2 (including Data Analysis) was used for patients’ data processing. Statistical analysis was conducted with Statistica software (version 8.0; StatSoft, Inc., Tulsa, OK, USA). Continuous variables were expressed as median values with an interquartile range (IQR: 25–75%). The Shapiro–Wilk and Kolmogorov–Smirnov tests were used to verify the data obtained after preliminary analysis and check the consistency of the group [20]. The Mann–Whitney U-test compared the distribution of continuous variables between different categories for independent samples (DTC/−T2DM group vs. DTC/+T2DM group). Pearson’s correlation coefficient (r) was used to assess the relationships between the measured variables. Significance was set at a *p*-value < 0.05.

## 3. Results

### 3.1. Characteristics of the Study Population

Clinical, hematological, and biochemical data are summarized in Table 1. There was no significant difference between the two groups (DTC/−T2DM and DTC/+T2DM) in terms of age. The median BMI of 29 kg/m^2^ shows that patients with DTC/−T2DM are overweight, while patients with DTC/+T2DM are obese (obesity class I) with a median BMI of 33.8 kg/m^2^ (*p* = 0.006) [21]. LVEF was lower in the patients with concomitant T2DM than in the group without T2DM (*p* < 0.001). Overt signs of heart failure, such as pulmonary congestion and peripheral edema, were absent at physical examination in all patients. Unexpectedly, the median cumulative dose of ^131^I given over 53 months average period was higher in female patients with DTC/+T2DM than in those without T2DM (*p* = 0.041). Levothyroxine treatment was prescribed to all patients enrolled in the study. There was no significant difference between the two groups regarding the median daily dose of levothyroxine. Highly significant differences in the absolute lymphocytes and platelets counts were observed among DTC/−T2DM and DTC/+T2DM patients (*p* = 0.015 and *p* = 0.002). Increased platelet count was reported in the DTC/+T2DM group [11]. Total cholesterol and lipids were comparable, with no statistical significance between the two groups. Hypercholesterolemia was the most prevalent cardiovascular risk factor, and its frequency did not differ between the two groups. Moreover, the absolute triglycerides count was higher in female patients with concomitant T2DM as a sign of diabetes (*p* = 0.049), and the serum ALP level was lower (*p* = 0.070). In summary, DTC female patients with concomitant T2DM received a cumulative dose of ^131^I higher than that received by female patients without T2DM and had a lower LVEF.

### 3.2. Correlations in DTC/−T2DM Group

**Figure 1:**
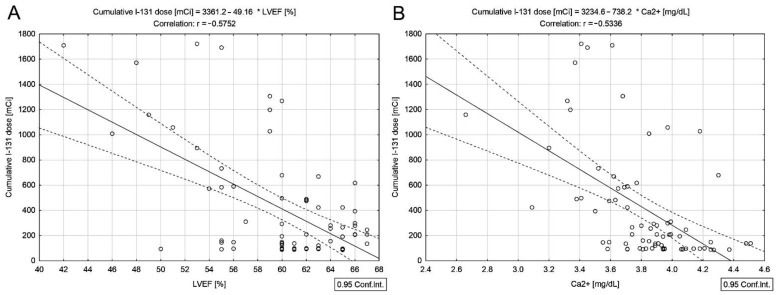
Correlations between the cumulative dose of ^131^I and LVEF (**A**), serum concentrations of Ca^2+^ (**B**) in differentiated thyroid cancer patients without type 2 diabetes mellitus.

In DTC/−T2DM patients, scatter plots shown in Figure 1 indicate an inverse relationship between cumulative ^131^I dose and LVEF (*r* = −0.57, *p* < 0.001) (Figure 1A) and serum Ca^2+^ (*r* = −0.53, *p* < 0.001) (Figure 1B).

**Figure 2:**
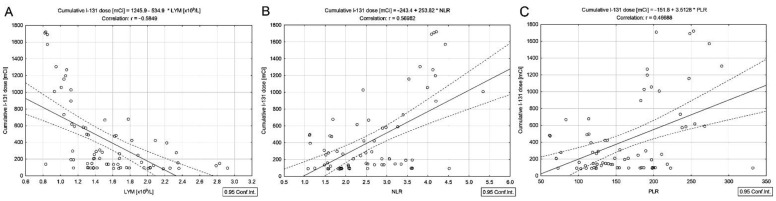
Correlations between the cumulative dose of ^131^I and absolute lymphocyte count (**A**), NLR (**B**), and PLR (**C**) in differentiated thyroid cancer patients without type 2 diabetes mellitus.

Moreover, the cumulative dose of ^131^I was negatively correlated with absolute lymphocyte count (*r* = −0.58, *p* < 0.001) (Figure 2A) and positively with NLR and PLR (*r* = 0.57 and *r* = 0.46, *p* < 0.001) (Figure 2B,C). No correlation between the cumulative ^131^I dose and the serum concentrations of total cholesterol, lipids, triglycerides, and ALP was noticed.

**Figure 3:**
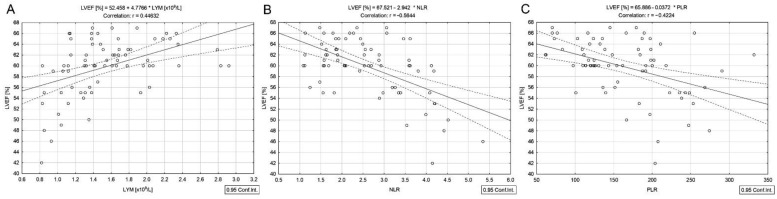
Correlations between LVEF and absolute lymphocyte count (**A**), NLR (**B**), and PLR (**C**) in differentiated thyroid cancer patients without type 2 diabetes mellitus.

However, the LVEF was positively correlated with absolute lymphocyte count (*r* = 0.44, *p* < 0.001) (Figure 3A) and serum Ca^2+^ (*r* = 0.29, *p* = 0.012) and negatively with NLR and PLR (*r* = −0.56 and *r* = −0.42, *p* < 0.001) (Figure 3B,C).

### 3.3. Correlations in DTC/+T2DM Group

**Figure 4:**
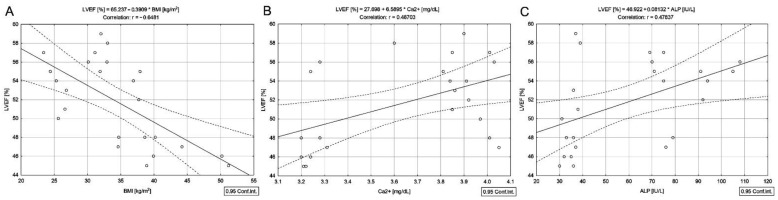
Correlations between LVEF and BMI (**A**), Ca^2+^ (**B**), and ALP (**C**) in differentiated thyroid cancer patients with type 2 diabetes mellitus.

**Figure 5:**
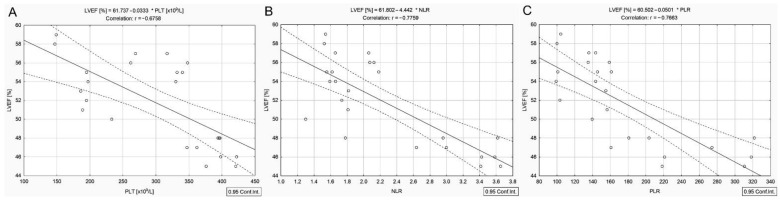
Correlations between LVEF and absolute platelet count (**A**), NLR (**B**), PLR (**C**) in differentiated thyroid cancer patients with type 2 diabetes mellitus.

In contrast to DTC/−T2DM patients, in the group with DTC associated with T2DM, no correlation between the cumulative dose of ^131^I and LVEF was observed. The cumulative dose of ^131^I was negatively correlated with serum concentrations of total cholesterol (*r* = −0.50, *p* = 0.012), lipids (*r* = −0.51, *p* = 0.011), triglycerides (*r* = −0.46, *p* = 0.022), ALP (*r* = −0.41, *p* = 0.047), absolute lymphocyte count (*r* = −0.55, *p* = 0.005), absolute neutrophil count (*r* = −0.45, *p* = 0.025), but it was not correlated with NLR and PLR. However, there was a negative correlation between LVEF and BMI (*r* = −0.64, *p* = 0.001) (Figure 4A), absolute neutrophil count (*r* = −0.54, *p* = 0.005), absolute platelet count (*r* = −0.67, *p* < 0.001) (Figure 5A), NLR (*r* = −0.77, *p* < 0.001) (Figure 5B), PLR (*r* = −0.76, *p* < 0.001) (Figure 5C) and positively with Ca^2+^ (*r* = 0.48, *p* = 0.016) (Figure 4B), ALP (*r* = 0.47, *p* = 0.018) (Figure 4C).

## 4. Discussion

The main findings of the present study are: (i) LVEF was lower in DTC/+T2DM than in DTC/−T2DM patients; (ii) LVEF was inversely correlated with cumulative ^131^I dose in the DTC/−T2DM group; (iii) platelet count was higher in DTC/+T2DM than in DTC/−T2DM patients; (iv) LVEF was inversely correlated with platelet count in the DTC/+T2DM group.

Ionizing radiation causes a significant reduction in blood cell counts in a dose-dependent manner [22,23,24]. Consequently, the targeted therapy with ^131^I can lead to long-term or temporary adverse effects, such as myelosuppression [22]. Hematological toxicity is a common adverse effect of ^131^I therapy [23,24]. Absolute blood lymphocyte and platelet counts measured in DTC/−T2DM patients were lower than in the DTC/+T2DM group (*p* = 0.015 and *p* = 0.002). The NLR value was higher and the PLR lower in DTC/−T2DM patients but without statistical significance, reflecting the immune status after cumulative radiotherapy. Because the peripheral blood samples were collected 6 months after the last ^131^I dose, the results do not reflect the transient ^131^I influence on bone marrow. However, high cumulative doses of ^131^I can cause significant long-term damage to hematopoietic system components, reducing blood counts. We believe that this is the first study to correlate NLR and PLR with cumulative ^131^I dosage in DTC/−T2DM vs. DTC/+T2DM patients. The cumulative ^131^I dose was negatively correlated with absolute lymphocyte count (Figure 2A) and positively with NLR and PLR (Figure 2B,C) in DTC/−T2DM patients. Rui et al. [24], comparing pre-^131^I therapy with 4–6 months post-^131^I therapy, showed that the number of treatment cycles and cumulative doses of ^131^I were associated with significant decline in lymphocyte and platelet counts. Radiation-induced lymphopenia and thrombocytopenia elucidate the positive correlation between high NLR and low PLR, and the cumulative dose administered to patients with DTC/−T2DM. The association of DTC with T2DM has led to a change in the tumor microenvironment [25]. The tumor microenvironment confers ionizing radiation with either immunosuppressive or immune-stimulating properties [10,26]. There is still a lack of information on the role and functionality of immune cells after irradiation. Chronic inflammation in T2DM is characterized by a prothrombotic state caused by the mutual activation of neutrophils and platelets [11]. Neutrophils may play a pro-tumor or anti-tumor role depending on the tumor microenvironment [11,13]. The inverse correlation between cumulative ^131^I dose and absolute neutrophil count (*r* = −0.45, *p* = 0.0025) confirms the results obtained by Wisdom et al. [13]. ^131^I affects early neutrophil infiltration, which is the first-line immune response, only in the DTC/+T2DM due to the altered tumor microenvironment resulting from the coexistence with T2DM. Moreover, as a confirmation of the altered tumor microenvironment in the presence of T2DM, no correlation between cumulative dose of ^131^I and absolute platelet count, NLR, and PLR was noticed.

The median LVEF measured in women with DTC/−T2DM was 60%. Even if the LVEF was within the reference range, the inverse correlation calculated between the cumulative dose of ^131^I and LVEF (Figure 1A) demonstrates an association between myocardial damage and the total radiation dose. The cardiac injury sustained from high cumulative doses will likely have a delayed onset, longer than our median monitoring period of 53 months. Kim et al. [27] showed no cumulative dose-dependent risk for cardiovascular disease in women with DTC with a median follow-up of 66 months. However, Kao et al. [28] concluded that even if ^131^I therapy was not associated with an increased risk of cardiovascular disease in DTC, cardiovascular surveillance is indicated in the patients receiving the cumulative dose of ^131^I above 100 mCi (3.7 GBq). The positive correlations between LVEF and absolute lymphocyte count (Figure 3A) and the negative correlations between LVEF and NLR and PLR (Figure 3B,C) confirm that radiation-related myocardial cell damage may result in the first phase in the activation of acute inflammatory cascades. NLR reflects the systemic change caused by radiotherapy [29]. At the same time, PLR reveals shifts in platelet and lymphocyte counts due to acute inflammatory and prothrombotic states [30]. It is well known that the history of heart failure is not linear because changes in the heart structure and function start long before the disease becomes clinically evident [31]. Radiation-induced cardiovascular disease is also described as a late effect in cancer patients treated with radiation therapy [32].

In the group of women with DTC coexisting with T2DM, there was no correlation between the cumulative dose of ^131^I and LVEF. LVEF was lower than in the group without T2DM (*p* < 0.001). It is well known that people with T2DM have a higher cardiovascular risk than those without [16]. The recent universal definition and classification of heart failure recognized T2DM as a prime risk factor for incident heart failure, suggesting individuals with T2DM as being in the first stage of heart failure (stage A) [33]. This study found that the absolute platelet and lymphocyte counts were higher in the DTC/+T2DM group than in DTC/−T2DM. Platelets are tiny blood cells with diverse vascular functions. Platelets in patients with T2DM are reported to exhibit hyper-reactivity due to external stimuli and undergo rapid consumption, leading to accelerated thrombopoiesis of more reactive platelets [34]. On the other hand, targeted ^131^I therapy can be considered an external stimulus that could significantly activate platelets in patients with DTC/+T2DM. However, the cumulative dose of ^131^I was not correlated with absolute platelet count in the DTC/+T2DM group. Still, it was slightly negatively correlated with the absolute neutrophil count. As mentioned before, chronic inflammation in T2DM is characterized by a prothrombotic state caused by the mutual activation of neutrophils and platelets [11]. We hypothesized that ^131^I therapy affects early neutrophil infiltration in the first phase. Neutrophils are essential mediators of radiation resistance [13]. Their infiltration could be the first-line immune response to ^131^I. Next, platelet number is increased by neutrophil secretion. Further, platelet–neutrophil complexes enhance platelet activation and thrombus formation. Inflammatory mediators released by activated platelets recruit more platelets and white blood cells to the site of inflammation in response to ^131^I, as evidenced by the increased level of platelets and lymphocytes in patients with DTC/+T2DM. Other factors that may contribute to increased platelet counts are dyslipidemia and obesity [34,35]. Patients with DTC/+T2DM have a higher triglycerides concentration than those with DTC/−T2DM (154.5 vs. 111.0 mg/dL). DTC/+T2DM female patients are obese, unlike those with DTC/−T2DM who are overweight (33.8 vs. 29 kg/m^2^). Platelets play a crucial role in the initiation and progression of diabetes-induced cardiovascular complications [34]. In T2DM, chronic and systemic inflammation is associated with abnormal clot formation (dysregulated coagulation) [35] and dysregulated inflammatory biomarkers, such as NLR and PLR. PLR has been closely related to T2DM and its chronic complications, cardiovascular disease, peripheral arterial disease, and tumors [36]. As a confirmation, our results showed a significantly higher median platelet count in the DTC/+T2DM group than in DTC/−T2DM (331 × 10^9^/L vs. 230 × 10^9^/L, *p* = 0.002) and a negative correlation between the platelet count and LVEF (Figure 5A). The negative correlation between LVEF and absolute platelet count (Figure 5A) and PLR (Figure 5C) demonstrates that cardiovascular risk in this group is due to a complex combination of various factors that seem to have a common history of both diabetes and cardiovascular disease rather than to the higher cumulative dose of ^131^I.

In plasma, calcium exists in three different forms: free or ionized calcium (Ca^2+^) accounting for 50% of total calcium; 45% plasma proteins-bound calcium; and 5% calcium complexed to bicarbonate, lactate, phosphate, and citrate [37]. The biological effect of calcium is determined by the amount of Ca^2+^, which is the only physiologically active form of calcium. A Ca^2+^ level lower than 3.8 mg/dL is considered an alarm signal in monitoring patients with DTC. In both study groups, the median level of Ca^2+^ was 3.8 mg/dL (Table 1). It is well known that Ca^2+^ is a major element in the electrical and contractile function of cardiomyocytes. Excitation–contraction coupling (ECC) is the process by which electrical stimulation results in the contraction of cardiac myofilaments, which involves sarcolemmal ion currents and various intracellular pathways. There was a positive correlation between Ca^2+^ and LVEF in both groups of patients (DTC/−T2DM: *r* = 0.29, *p* = 0.012 and DTC/+T2DM: *r* = 0.48, *p* = 0.016). The correlation was stronger in the DTC/+T2DM group than in the DTC/−T2DM, probably because of the T2DM features. Our results are in accordance with those obtained by Wang et al. [38], who noted that low serum calcium was associated with left ventricular systolic dysfunction in a Chinese population represented by 5938 patients with coronary artery disease. On the other hand, our finding showed that a decrease in Ca^2+^ level, not an increase, was associated with decreased LVEF in patients with T2DM, as published by Li et al. [39]. The explanation is that the DTC/+T2DM patients enrolled in our study have hypocalcemia. Even though they are on the calcium replacement therapy, the Ca^2+^ concentration did not exceed 3.8 mg/dL. Considering the roles of Ca^2+^ in coupling myocardial excitation–contraction and cardiac electrophysiological effect, we believe that low Ca^2+^ may make sense in the prognosis among patients with DTC/+T2DM with a median LVEF of 52.5%. It is possible that low Ca^2+^ levels may affect myocardial contractility in patients with DTC/+T2DM. However, the causal relationship between low Ca^2+^ and LVEF remains to be elucidated and requires further study.

ALP is a circulating enzyme primarily derived from bone and liver. Previous studies have shown an association between ALP as an inflammatory mediator and cardiovascular events [40]. The results of the studies on the serum level of ALP in patients with T2DM are contradictory (higher, lower, or similar to that of the control group) and do not exist in those with T2DM coexisting with DTC. Dutta et al. [41] showed that the serum concentration of ALP was lower in patients with T2DM than in those without T2DM (88.5 ± 33.3 IU/L vs. 214.7 ± 59.7 IU/L, *p* ˂ 0.001). In our study, the ALP serum level was lower in the DTC/+T2DM group than in the DTC/−T2DM group but without statistical significance (54.0 IU/L vs. 66.5 IU/L). ALP is less known for its ability to reduce inflammation by dephosphorylating triggering moieties, such as bacterial lipopolysaccharides and extracellular nucleotides [42]. A prominent feature of T2DM is chronic and systemic inflammation. Low ALP levels in the DTC/+T2DM group could be due to the T2DM specific inflammation. The linear association between LVEF and ALP (Figure 4C), which has a key role in maintaining and restoring physiological barriers that may become hyperpermeable and/or dysfunctional during systemic ischemia and inflammation [43], proves once again that cardiovascular risk in the DTC/+T2DM group is due to the mechanisms underlying the pathophysiology of T2DM and not to the higher cumulative dose of ^131^I.

This study has certain limitations. The most apparent weakness is related to the small number of patients enrolled. However, despite the small sample size, the population was homogenous and included only women. Moreover, the two study groups matched in terms of age. Second, the present results were from a monitoring period of only 53 months. Future studies should focus on long-term outcomes. Other weaknesses and limitations of this study are the lack of longitudinal or comparative data in enrolled patients to conclude the “cumulative effect” or “progressive effect” of ^131^I. Longitudinal data after each ^131^I cycle in individual patients are the most accurate and valid measure of cumulative effect. However, despite this weakness, a large-dose effect of ^131^I on echocardiographic indices of left ventricular function and peripheral blood parameters was investigated. The comparison of pre-^131^I therapy and post-^131^I therapy data for the same subject may demonstrate the progressive effect of radiation-associated cardiovascular toxicity. A future study will focus on this subject.

In summary, the present study results demonstrated an inverse relationship between LVEF and high cumulative doses of ^131^I in patients with DTC/−T2DM. This association does not exist in the DTC/+T2DM group. LVEF was lower in DTC/+T2DM than in DTC/−T2DM patients. LVEF was inversely correlated with increased platelet count in the DTC/+T2DM group. Further investigation is required to confirm these findings.

## Figures and Tables

**Table 1:** Clinical, hematological, and biochemical data in the study groups.

Variables	DTC/−T2DM	DTC/+T2DM	*p*-Value
	*n* = 72	*n* = 24	
Age (years) ^a^	57.9 ± 8.7	61.1 ± 7.2	0.147
BMI (kg/m^2^) ^b^	29.0 (26.2–33.4)	33.8 (28.4–38.7)	0.006
LVEF (%) ^b^	60.0 (56.5–63.5)	52.5 (47.5–55.5)	<0.001
Cumulative ^131^I dose (mCi) ^b^ Levothyroxine dose (mcg/day)	208.5 (152.8–577.0) 107.4 (86.2–149.5)	494.0 (176.0–817.0) 107.2 (83.4–143.8)	0.041 0.231
Lymphocytes (×10^9^/L) ^b^	1.4 (1.1–1.8)	1.9 (1.4–2.1)	0.015
Neutrophils (×10^9^/L) ^b^	3.7 (3.0–4.4)	3.6 (3.1–4.5)	0.731
Platelets (×10^9^/L) ^b^	230.0 (193.0–279.5)	331.0 (196.0–385.0)	0.002
NLR ^b^	2.5 (1.8–3.4)	1.9 (1.6–2.9)	0.150
PLR ^b^	152.9 (119.3–199.1)	155.6 (135.4–210.8)	0.629
Total Cholesterol (mg/dL) ^b^	277.0 (215.5–350.0)	272.0 (149.5–332.5)	0.265
Lipids (mg/dL) ^b^	851.5 (688.0–1057.0)	852.0 (682.0–1045.5)	0.479
Triglycerides (mg/dL) ^b^	111.0 (94.0–170.0)	154.5 (102.5–272.0)	0.049
Ca^2+^ (mg/dL) ^b^	3.8 (3.6–3.9)	3.8 (3.2–3.9)	0.087
ALP (IU/L) ^b^	66.5 (54.0–82.0)	54.0 (36.0–77.5)	0.070

ALP, alkaline phosphatase; BMI, body mass index; Ca^2+^, ionized calcium; DTC/−T2DM, differentiated thyroid cancer without type 2 diabetes mellitus; DTC/+T2DM, differentiated thyroid cancer associated with type 2 diabetes mellitus; LVEF, left ventricular ejection fraction; NLR, neutrophil-to-lymphocyte ratio; PLR, platelet-to-lymphocyte ratio; ^131^I, radioiodine; ^a^ mean ± standard deviation; ^b^ Data are expressed as median and interquartile ranges (25–75%).

## Data Availability

The data presented in this study are available on request from the corresponding author.

## References

[1] International Diabetes Federation IDF Diabetes Atlas.

[2] Ling S., Brown K., Miksza J.K., Howells L., Morrison A., Issa E., Yates T., Khunti K., Davies M.J., Zaccardi F. (2020). Association of Type 2 Diabetes With Cancer: A Meta-analysis With Bias Analysis for Unmeasured Confounding in 151 Cohorts Comprising 32 Million People. Diabetes Care.

[3] Ling S., Brown K., Miksza J.K., Howells L.M., Morrison A., Issa E., Yates T., Khunti K., Davies M.J., Zaccardi F. (2021). Risk of cancer incidence and mortality associated with diabetes: A systematic review with trend analysis of 203 cohorts. Nutr. Metab. Cardiovasc. Dis..

[4] Roh E., Noh E., Hwang S.Y., Kim J.A., Song E., Park M., Choi K.M., Baik S.H., Cho G.J., Yoo H.J. (2021). Increased Risk of Type 2 Diabetes in Patients With Thyroid Cancer After Thyroidectomy: A Nationwide Cohort Study. J. Clin. Endocrinol. Metab..

[5] Kalra S., Aggarwal S., Khandelwal D. (2019). Thyroid Dysfunction and Type 2 Diabetes Mellitus: Screening Strategies and Implications for Management. Diabetes Ther..

[6] Fugazzola L., Elisei R., Fuhrer D., Jarzab B., Leboulleux S., Newbold K., Smit J. (2019). 2019 European Thyroid Association Guidelines for the Treatment and Follow-Up of Advanced Radioiodine-Refractory Thyroid Cancer. Eur. Thyr. J..

[7] Haugen B.R., Alexander E.K., Bible K.C., Doherty G.M., Mandel S.J., Nikiforov Y.E., Pacini F., Randolph G.W., Sawka A.M., Schlumberger M. (2016). 2015 American Thyroid Association Management Guidelines for Adult Patients with Thyroid Nodules and Differentiated Thyroid Cancer: The American Thyroid Association Guidelines Task Force on Thyroid Nodules and Differentiated Thyroid Cancer. Thyroid.

[8] la Cour J.L., Hedemann-Jensen P., Søgaard-Hansen J., Nygaard B., Jensen L.T. (2013). Modeling the absorbed dose to the common carotid arteries following radioiodine treatment of benign thyroid disease. Ann. Nucl. Med..

[9] la Cour J.L., Andersen U.B., Sørensen C.H., Nygaard B., Jensen L.T. (2016). Radioiodine Therapy Does Not Change the Atherosclerotic Burden of the Carotid Arteries. Thyroid.

[10] Gheorghe D., Stanciu M., Zamfirescu A., Stanciu A. (2021). TNF-α May Exert Different Antitumor Effects in Response to Radioactive Iodine Therapy in Papillary Thyroid Cancer with/without Autoimmune Thyroiditis. Cancers.

[11] Herrero-Cervera A., Soehnlein O., Kenne E. (2022). Neutrophils in chronic inflammatory diseases. Cell. Mol. Immunol..

[12] Kuravi S.J., Harrison P., Rainger G.E., Nash G.B. (2019). Ability of Platelet-Derived Extracellular Vesicles to Promote Neutrophil-Endothelial Cell Interactions. Inflammation.

[13] Wisdom A.J., Hong C.S., Lin A.J., Xiang Y., Cooper D.E., Zhang J., Xu E.S., Kuo H.-C., Mowery Y.M., Carpenter D.J. (2019). Neutrophils promote tumor resistance to radiation therapy. Proc. Natl. Acad. Sci. USA.

[14] Marques P., de Vries F., Dekkers O.M., Korbonits M., Biermasz N.R., Pereira A.M. (2021). Serum Inflammation-based Scores in Endocrine Tumors. J. Clin. Endocrinol. Metab..

[15] D’Emic N., Engelman A., Molitoris J., Hanlon A., Sharma N.K., Moeslein F.M., Chuong M.D. (2016). Prognostic significance of neutrophil-lymphocyte ratio and platelet-lymphocyte ratio in patients treated with selective internal radiation therapy. J. Gastrointest. Oncol..

[16] Roman G., Pantea Stoian A., Stoian A.P. (2021). Cardiovascular risk/disease in type 2 diabetes mellitus. Type 2 Diabetes: From Pathophysiology to Cyber Systems.

[17] Sisson J.C., Freitas J., McDougall I.R., Dauer L., Hurley J.R., Brierley J.D., Edinboro C.H., Rosenthal D., Thomas M.J., Wexler J.A. (2011). Radiation Safety in the Treatment of Patients with Thyroid Diseases by Radioiodine 131I: Practice Recommendations of the American Thyroid Association, The American Thyroid Associ-ation Taskforce on Radioiodine Safety. Thyroid.

[18] Bedel C., Korkut M., Armağan H.H. (2020). NLR, d-NLR and PLR can be affected by many factors. Int. Immunopharmacol..

[19] Jacobse J.N., Steggink L., Sonke G.S., Schaapveld M., Hummel Y.M., Steenbruggen T.G., Lefrandt J.D., Nuver J., Crijns A.P., Aleman B.M. (2019). Myocardial dysfunction in long-term breast cancer survivors treated at ages 40–50 years. Eur. J. Heart Fail..

[20] Stanciu A.E., Zamfir-Chiru-Anton A., Stanciu M.M., Pantea-Stoian A., Nitipir C., Gheorghe D.C. (2020). Serum melatonin is inversely associated with matrix metalloproteinase-9 in oral squamous cell carcinoma. Oncol. Lett..

[21] Weir C.B., Jan A. (2022). BMI Classification Percentile and Cut Off Points.

[22] Prinsen H.T., Hesselink E.N.K., Brouwers A.H., Plukker J.T.M., Sluiter W.J., van der Horst-Schrivers A.N.A., van Imhoff G.W., Links T.P. (2015). Bone Marrow Function After^131^I Therapy in Patients With Differentiated Thyroid Carcinoma. J. Clin. Endocrinol. Metab..

[23] Hu T., Meng Z., Zhang G., Jia Q., Tan J., Zheng W., Wang R., Li X., Liu N., Zhou P. (2016). Influence of the first radioactive iodine ablation on peripheral complete blood count in patients with differentiated thyroid cancer. Medicine.

[24] Rui Z., Wu R., Zheng W., Wang X., Meng Z., Tan J. (2021). Effect of ¹³¹I Therapy on Complete Blood Count in Patients with Differentiated Thyroid Cancer. Med. Sci. Monit..

[25] Shlomai G., Neel B., Leroith D., Gallagher E.J. (2016). Type 2 Diabetes Mellitus and Cancer: The Role of Pharmacotherapy. J. Clin. Oncol..

[26] Xue H., Qiu B., Wang H., Jiang P., Sukocheva O., Fan R., Xue L., Wang J. (2021). Stereotactic Ablative Brachytherapy: Recent Advances in Optimization of Radiobiological Cancer Therapy. Cancers.

[27] Kim K.J., Song J.E., Kim J.Y., Bae J.H., Kim N.H., Yoo H.J., Kim H.Y., Seo J.A., Kim N.H., Lee J. (2020). Effects of radioactive iodine treatment on cardiovascular disease in thyroid cancer patients: A nationwide cohort study. Ann. Transl. Med..

[28] Kao C.-H., Chung C.-H., Chien W.-C., Shen D.H.-Y., Lin L.-F., Chiu C.-H., Cheng C.-Y., Sun C.-A., Chang P.-Y. (2021). Radioactive Iodine Treatment and the Risk of Long-Term Cardiovascular Morbidity and Mortality in Thyroid Cancer Patients: A Nationwide Cohort Study. J. Clin. Med..

[29] Yoon C.I., Kim D., Ahn S.G., Bae S.J., Cha C., Park S., Park S., Kim S.I., Lee H.S., Park J.Y. (2020). Radiotherapy-Induced High Neutrophil-to-Lymphocyte Ratio is a Negative Prognostic Factor in Patients with Breast Cancer. Cancers.

[30] Gasparyan A.Y., Ayvazyan L., Mukanova U., Yessirkepov M., Kitas G.D. (2019). The Platelet-to-Lymphocyte Ratio as an Inflammatory Marker in Rheumatic Diseases. Ann. Lab. Med..

[31] Stanciu A.E. (2019). Cytokines in heart failure. Adv. Clin. Chem..

[32] Nielsen K.M., Offersen B.V., Nielsen H.M., Vaage-Nilsen M., Yusuf S.W. (2017). Short and long term radiation induced cardiovascular disease in patients with cancer. Clin. Cardiol..

[33] Ceriello A., Catrinoiu D., Chandramouli C., Cosentino F., Dombrowsky A.C., Itzhak B., Lalic N.M., Prattichizzo F., Schnell O., Seferović P.M. (2021). Heart failure in type 2 diabetes: Current perspectives on screening, diagnosis and management. Cardiovasc. Diabetol..

[34] Rodriguez B.A.T., Johnson A.D. (2020). Platelet Measurements and Type 2 Diabetes: Investigations in Two Population-Based Cohorts. Front. Cardiovasc. Med..

[35] Randeria S., Thomson G.J.A., Nell T.A., Roberts T., Pretorius E. (2019). Inflammatory cytokines in type 2 diabetes mellitus as facilitators of hypercoagulation and abnormal clot formation. Cardiovasc. Diabetol..

[36] Johny E., Bhaskar P., Alam J., Kuladhipati I., Das R., Adela R. (2021). Platelet Mediated Inflammation in Coronary Artery Disease with Type 2 Diabetes Patients. J. Inflamm. Res..

[37] Gonçalves J.A.F., Costa T., Rema J., Pinto C., Magalhães M., Esperança A., Sousa L. (2019). Hypocalcemia in cancer patients: An exploratory study. Porto Biomed. J..

[38] Wang Y., Ma H., Hao X., Yang J., Chen Q., Lu L., Zhang R. (2016). Low serum calcium is associated with left ventricular systolic dysfunction in a Chinese population with coronary artery disease. Sci. Rep..

[39] Li J., Wu N., Dai W., Jiang L., Li Y., Li S., Wen Z. (2016). Association of serum calcium and heart failure with preserved ejection fraction in patients with type 2 diabetes. Cardiovasc. Diabetol..

[40] Kunutsor S.K., Apekey T.A., Khan H. (2014). Liver enzymes and risk of cardiovascular disease in the general population: A meta-analysis of prospective cohort studies. Atherosclerosis.

[41] Dutta M., Pakhetra R., Garg M. (2012). Evaluation of bone mineral density in type 2 diabetes mellitus patients before and after treatment. Med. J. Armed Forces India.

[42] Kats S., Brands R., Seinen W., de Jager W., Bekker M.W.A., Hamad M.A.S., Tan M.E.S.H., Schonberger J.P.A.M. (2009). Anti-Inflammatory Effects of Alkaline Phosphatase in Coronary Artery Bypass Surgery with Cardiopulmonary Bypass. Recent Pat. Inflamm. Allergy Drug Discov..

[43] Presbitero A., Mancini E., Brands R., Krzhizhanovskaya V.V., Sloot P.M.A. (2018). Supplemented Alkaline Phosphatase Supports the Immune Response in Patients Undergoing Cardiac Surgery: Clinical and Computational Evidence. Front. Immunol..

